# Molecular Regulation of Histamine Synthesis

**DOI:** 10.3389/fimmu.2018.01392

**Published:** 2018-06-20

**Authors:** Hua Huang, Yapeng Li, Jinyi Liang, Fred D. Finkelman

**Affiliations:** ^1^The Department of Biomedical Research, National Jewish Health, Denver, CO, United States; ^2^The Department of Immunology and Microbiology, University of Colorado School of Medicine, Aurora, IL, United States; ^3^Department of Parasitology, Zhongshan School of Medicine, Sun Yat-sen University, Guangzhou, China; ^4^The Division of Immunobiology, Cincinnati Children’s Hospital Medical Center, Cincinnati, OH, United States; ^5^The Division of Immunology, Allergy and Rheumatology, Department of Medicine, University of Cincinnati College of Medicine, Cincinnati, OH, United States

**Keywords:** histamine, histidine decarboxylase, enhancers, promoter, gene regulation

## Abstract

Histamine is a critical mediator of IgE/mast cell-mediated anaphylaxis, a neurotransmitter and a regulator of gastric acid secretion. Histamine is a monoamine synthesized from the amino acid histidine through a reaction catalyzed by the enzyme histidine decarboxylase (HDC), which removes carboxyl group from histidine. Despite the importance of histamine, transcriptional regulation of *HDC* gene expression in mammals is still poorly understood. In this review, we focus on discussing advances in the understanding of molecular regulation of mammalian histamine synthesis.

## Introduction

Bill Paul’s impact on immunology is broad and enormous. Like many of his former trainees, I had the good fortune to learn from him. Bill’s mentorship has nurtured my lifelong interest in studying type 2 immune responses that cause allergic diseases and protect against parasitic infections. In the early years of my laboratory, we had investigated how naïve CD4^+^ T cells commit into T helper type 1 cells by silencing the potential to transcribe the *Il4* gene (1–3). More recently, we extended our efforts to understand how a bi-potential basophil and mast cell progenitor acquires the capacity to transcribe a set of basophil-specific or mast cell-specific genes while simultaneously repressing transcription of a gene set that is specific for the other cell type (4). With a newly gained understanding of a network of transcription factors and their targeted enhancers (5), our laboratory has chosen to investigate the *Hdc* gene (encode histidine decarboxylase, a rate-limiting enzyme for histamine synthesis) in greater detail.

Anaphylaxis is a serious allergic reaction that is rapid in onset and can be life threatening. The clinic manifestations include symptoms that involve the skin, gastrointestinal track, respiratory system, and cardiovascular system (6). Anaphylaxis can be caused by allergy to foods, insect venoms, medications, and other agents (6). The incidence of food-induced anaphylaxis has risen dramatically in developed countries during the past several decades (7–9). The cost of treating food allergy is estimated at about 25 billion dollars annually in the US alone (10).

**Figure 1:**
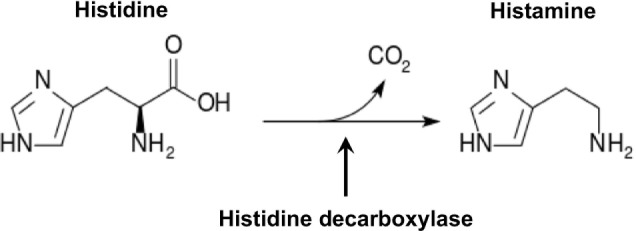
Histamine synthesis.

Histamine plays an essential role in IgE-medicated anaphylaxis, the most common type of anaphylaxis (11–14). Histamine was first purified from ergot fungi (15) in 1910 and from human tissues (16) in 1927. Histamine has pleiotropic effects on skin and the cardiovascular, respiratory, digestive, central nervous, and immune systems (17). It is a profound vasodilator that increases blood vessel permeability, allowing blood leukocytes to enter tissues to promote inflammatory responses. Relatively large quantities of histamine can cause a rapid decrease in body temperature due to massive leakage of blood plasma into the extravascular space. Rapid release of large amounts of histamine leads to anaphylaxis (12, 14). Histamine belongs to a family of biogenic amines that includes neurotransmitters, such as serotonin and dopamine, and hormones, such as epinephrine. Biogenic amines that contain one or more amine groups are formed mainly by decarboxylation of amino acids. Histamine is a monoamine synthesized from the amino acid histidine through a reaction catalyzed by the enzyme histidine decarboxylase (HDC), which removes carboxyl group from histidine (Figure 1). Although histamine can be synthesized by bacteria found in contaminated food (18) and in the gut of asthma patients (17, 19), in this review, we focus on discussing advances in the understanding of molecular regulation of mammalian histamine synthesis.

## Histamine-Producing Cells in Mammals and Stimuli that Trigger Histamine Release

**Figure 2:**
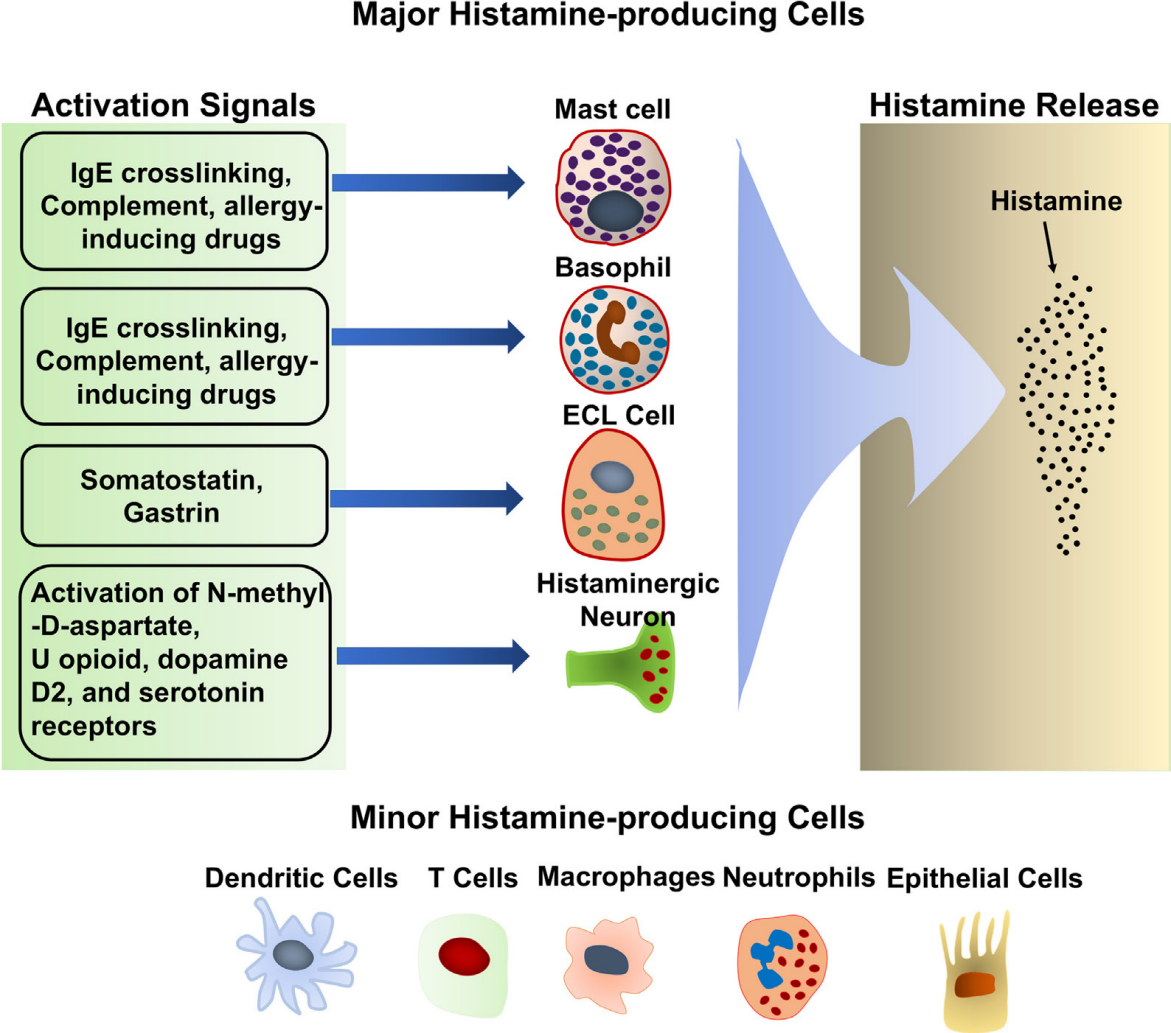
Histamine-producing cells and stimuli that trigger histamine release.

Histamine is synthesized primarily by mast cells, basophils, histaminergic neurons in the basal ganglia of the brain and enterochromaffin-like cells (ECL) in the stomach. These cells produce large amounts of histamine and are thought to be the major histamine-producing cells (Figure 2). They continuously synthesize histamine, which is then linked to the carboxyl group of heparin and stored in intracellular granules until the cells receive the appropriate activating stimulus. Upon external stimulation, these cells degranulate, releasing the stored histamine. Stimuli that trigger histamine release by these major histamine-producing cells have been reviewed extensively (20–25). Antigen crosslinking of antigen-specific IgE bound to the high-affinity IgE receptor, FcεRI, on the mast cell and basophil surface is the most robust stimulus that triggers histamine release by these cells (20–23). Substance P and allergy-inducing drugs that bind to G-protein-coupled receptors can also trigger basophils and mast cells to release histamine *via* different signaling pathway (23, 26). In addition, complement components, such as the C3a and C5a “anaphylatoxins,” have also been shown to induce histamine release by mast cells (27). Many cytokines, including IL-3, IL-18, IL-33, GM-CSF, and SCF, promote histamine synthesis (28–30). In general, cytokines alone do not induce histamine release although it remains controversial whether IL-33 can have this effect. Some reports describe that IL-33 stimulates histamine release (31, 32), while other reports dispute this (33, 34). It is suggested that IL-33 alone does not induce histamine release by basophils, but enhances histamine release in response to IgE/FcεRI crosslinking (35).

Additional histamine-producing cells have also been identified, including T cells (36), dendritic cells (37), macrophages (38, 39), and epithelial cells (40, 41) (Figure 2). In contrast to mast cells and basophils, these cells produce relative small quantities of histamine and do not store it in their cytoplasm (42). The small amounts of histamine that they produced are released without external stimulation (42). The biological significance of the small amounts of histamine produced by these minor histamine-producing cells remains unclear. Cell type-specific deletion of the *Hdc* gene, which encodes HDC, an enzyme essential for histamine synthesis, would shed light on the role of histamine synthesis and secretion by the minor histamine-producing cells.

## HDC and Histamine Synthesis in Mammals

After several groups purified mammalian HDC protein from fetal rat liver and mouse mastocytoma P-815 cells (43–45), a cDNA that encodes this protein was subsequently cloned (46, 47). The *Hdc* gene encodes HDC protein, which has a molecular mass of 74 kDa and is a proenzyme with little or no enzyme activity. Once the proenzyme is cleaved at the site near its c-terminus, presumably by Caspase-9, it yields a 53 kDa N-terminal and a 20 kDa C-terminal subunit. The 20 kDa C-terminal subunit is believed to possess inhibitory activity (48). The 53 kDa N-terminal subunit forms a homodimer that is an active decarboxylase (48, 49). HDC is the primary enzyme that catalyzes histamine synthesis. Mice deficient in the *Hdc* gene fail to synthesize histamine and have reduced or absent IgE-mediated anaphylactic responses (50–53). Several potent HDC inhibitors have been identified, including the histidine derivatives α-fluoromethyl histidine, histidine methyl ester, and pirodoxal histidine methyl ester (54–56). However, these HDC inhibitors have not been further developed for clinical use.

## *HDC* Gene Expression and Histamine Synthesis in Basophils and Mast Cells

*Hdc* gene expression and histamine synthesis are regulated both positively and negatively by a range of factors. Notably, crosslinking of FcεRI by antigen binding to FcεRI-associated IgE increases mast cell *Hdc* mRNA expression and histamine synthesis (57, 58). These mast cell activation-induced increases in *Hdc* mRNA expression and histamine synthesis are also induced by phorbol 12-myristate 13-acetate (59). *Hdc* mRNA expression and histamine synthesis also increase as immature mast cells undergo maturation. Bone marrow-derived mast cells (BMMCs) appear immature because they contain relatively little histamine and express relatively low levels of FcεRI (60). These immature mast cells develop into mature mast cells with higher amounts of histamine *in vivo* if they are adoptively transferred into the peritoneal cavity (61). However, it is not clear if *in vivo* exposure to IgE promotes maturation and increases *Hdc* mRNA expression.

In this regard, we demonstrated that chlorotoxin, which induces mast cell maturation (62), strongly upregulates *Hdc* gene expression in BMMCs within few hours after the treatment (5). The mechanism by which chlorotoxin enhances *Hdc* gene transcription remains to be determined. It is conceivable that chlorotoxin activates mast cells by binding to an acidic glycosphingolipid, ganglioside G, that has been shown to be expressed on the mast cell surface (62). Chlorotoxin-triggered signals in mast cells then activate transcription factors that directly and rapidly promote *Hdc* gene transcription. It is unknown whether bacteria in the gut of allergic patients can promote *Hdc* mRNA and histamine synthesis by producing substances similar to chlorotoxin.

In line with the notion that factors promoting mast cell maturation also enhance histamine synthesis, cytokines that promote basophil and mast cell maturation, such as IL-3, IL-18, IL-33, GM-CSF, and SCF, have also been reported to increase HDC activity (28–30, 63). It is unclear whether these cytokines regulate *Hdc* gene transcription by increasing the expression of the genes that encode *Hdc* gene-activating transcription factors or by activating already expressed transcription factors to induce transcription of the *Hdc* gene. Other substances, including chemokines, neuropeptide substance P, and IL-1α have also been reported to induce *Hdc* mRNA and histamine synthesis (64, 65).

By contrast, mitochondrial uncoupling protein 2, a mitochondrial transporter protein that transfers anions from the inner to the outer mitochondrial membrane and protons from the outer to the inner mitochondrial membrane, inhibits *Hdc* mRNA expression and histamine synthesis, possibly by suppressing the production of reactive oxygen species (66). Substances found in fruits and vegetables, such as quercetin (67), and in green tea, such as epigallocatechin gallate, also potently inhibit HDC (68). More detailed examination of negative regulators of *Hdc* mRNA expression should promote development of agents that may be able to prevent and treat food allergy and other histamine-mediated allergic inflammatory disorders.

**Figure 3:**
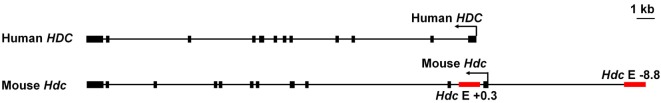
Genomic structures of the human and mouse histidine decarboxylase (*HDC*) gene. Red bars indicate the enhancers we described.

The human *HDC* gene is located in the 15q21.2 region of chromosome 15. It contains 12 exons (69) (Figure 3). Eight predicted isoforms can be generated by alternative splicing and two actual isoforms have been described (70). *HDC* mRNA is expressed broadly in many organs, with the highest expression levels found in the gallbladder, stomach, and lung (71). Because the RNA-seq data for normal tissues in the Human Protein Atlas were obtained from intact tissues, it is not clear whether the human *HDC* gene is expressed predominantly in known histamine-producing cells, such as mast cells and ECL in high *HDC*-expressing tissues, or predominantly in other cell types in those tissues. In contrast to the human *Hdc* gene, the mouse *HDC* gene is located in chromosome 2 (72). It resembles the human gene in that it contains 12 exons, is expressed broadly in many tissues with the highest expression levels in lung, ovary, and subcutaneous fat pads (72, 73), and is 86% homologous with the human gene (https://www.ncbi.nlm.nih.gov/homologene/20490); however, there are only three predicted isoforms and no isoform, other than the classical one, have been found for murine *Hdc* (72).

There is still limited knowledge of how *Hdc* gene expression is regulated transcriptionally. Most previous work has concentrated on the promoter region of this gene. Deletion analysis of *Hdc* promoter-driven luciferase reporter gene transcription demonstrated that the transcription factor SP1 binds to a GC box (GGGGCGGGG) found in both the human and mouse *Hdc* gene promoters (72, 74). Several promoter elements have been reported to negatively regulate *Hdc* gene transcription. For example, the transcription factors YY1 and KLF4 have been shown to negatively regulate the *Hdc* gene by suppressing SP1 in a gastric cancer cell line (75, 76).

By contrast, *Hdc* gene expression is positively regulated by the transcription factor GATA binding protein 2 (GATA2), a member of the GATA family of transcription factors. GATA2 is critical for survival and proliferation of hematopoietic stem cells (77, 78), granulocyte-monocyte progenitor differentiation (79), and basophil and mast cell differentiation (80, 81) and is required for connective tissue mast cell development (5). By contrast, basophil development is not affected in connective tissue-specific *Gata2*-deficient mice (5). We have also found that mucosal and connective tissue-specific *Gata2*-deficient mice fail to develop both mucosal and connective tissue mast cells, indicating that GATA2 is required for both mucosal and connective tissue mast cell development (Li et al., unpublished data). To distinguish the role of GATA2 in regulating the *Hdc* gene from its role in mast cell development, we used an inducible gene deletion method to delete the *Gata2* gene from mast cells after they had fully differentiated. In this inducible gene deletion model, the enzyme Cre is fused to the estrogen receptor (ER) and the ER-Cre fusion product is induced to enter the cell nucleus to cleave a floxed gene of interest by the ER ligand 4-hydroxytamoxifen (82). Using this method, we demonstrated that GATA2 plays a critical role in regulating *Hdc* gene expression in even fully differentiated mast cells. However, in contrast to its role in mast cell development, GATA2 is not needed for survival of fully differentiated mast cells (83).

More recently, our group has used active histone mark ChIP and reporter gene transcription assays to identify and characterize two *Hdc* enhancers in mast cells. Epigenomic studies demonstrate that monomethylation of lysine 4 on histone 3 (H3K4me1) marks genes that are poised to be transcribed, whereas acetylation of lysine 27 on histone 3 (H3K27ac) identifies genes that are actively being transcribed. The combined presence of H3K4me1 and H3K27ac modifications predicts enhancer activity (84–88). Our H3K4me1 and H3K27ac ChIP-seq analysis of BMMCs identified two putative *Hdc* enhancers located −8.8 kb upstream and +0.3 kb downstream from the transcription start site of the *Hdc* gene (Figure 3). We demonstrated that the −8.8 kb *Hdc* enhancer, but not the +0.3 kb *Hdc* enhancer, increases minimal *Hdc* promoter activity in a luciferase reporter gene transcription assay. The transcription factor MITF binds to the −8.8 *Hdc* enhancer and drives its enhancer activity. Indeed, MITF overexpression largely restores *Hdc* gene expression in *Gata2*-deficient mast cells. Our study also suggests that GATA2 induces MITF and that these two transcription factors together direct full *Hdc* gene transcription in mast cells in a feed-forward manner. However, it is not certain that the −8.8 kb *Hdc* enhancer is fully responsible for positive regulation of the *Hdc* gene, because *in vivo* importance of the +0.3 kb *Hdc* enhancer in *Hdc* gene transcription cannot be ruled out by the luciferase reporter gene transcription assay alone (5).

Despite remarkable progress in genome-wide annotation of potential enhancers, functional validation of annotated enhancers remains an unmet challenge. Transgenic mice, reporter gene assay, and CRISPR/Cas9 genome editing have been used to validate the biological functions of enhancers identified by histone marks. Each of these methods has its strengths and weaknesses (89, 90). The reporter gene assay has been widely used to assess enhancer activity. It is simple, rapid, and efficient at assessing promoter and enhancer activity in transiently or stably transfected cell lines. The limitation of the transient reporter gene assay is that it does not measure promoter and enhancer activity in the context of chromatin. Despite this disadvantage, this reductionist approach is useful for assessing binding of transcription factors to *cis* regulatory elements in accessible regions. It has been reported that ~60% of annotated enhancers show enhancer activity by the luciferase reporter gene assay (86, 91–94). The *in vivo* function of the −8.8 *Hdc* enhancer requires further investigation.

## Histamine Synthesis in the Central Nervous System and the Stomach

In addition to its activity as a vasoactive mediation, histamine is a neurotransmitter and a regulator of gastric acid secretion. *HDC* mRNA is expressed in the brain exclusively in the basal ganglia (95). Specific ablation of histaminergic neurons leads to repetitive movements (96), that resemble the signs of Tourette syndrome (97). Consistent with this, a nonsense mutation at the human *HDC* gene (W317X) has been identified in a family of patients with this syndrome (97, 98) and mice completely deficient in *Hdc* gene transcription develop a Tourette-like syndrome (97, 99). However, the mechanisms involved in *Hdc* gene regulation in the basal ganglia are currently unknown. In the stomach, histamine is synthesized in ECL and is released from these cells upon gastrin and acetylcholine stimulation. The released histamine then stimulates parietal cells to secrete stomach acid (25, 100). Mice deficient in the *Hdc* gene fail to fully acidify their gastric contents (100), which can lead to indigestion, diarrhea, constipation, or rectal itching (101). Clinically, histamine 2 (H_2_) receptor antagonists, such as ranitidine, are currently used to ameliorate stomach hyperacidity and peptic ulcer disease by blocking this receptor on the hydrochloric acid-producing parietal cells in the stomach (102). At present, it is not known how the *Hdc* gene is regulated in ECL. It is most likely that different transcription factors are used to regulate the *Hdc* gene in basal ganglia and ECL.

## Concluding Remarks

Histidine decarboxylase is the rate-limiting enzyme for histamine synthesis. Understanding transcriptional regulation of the *Hdc* gene will advance our knowledge about how this gene detects extracellular stimuli and increases its transcription, leading to histamine synthesis, replenishment, and accumulation that exacerbate allergic inflammation and anaphylaxis. Fine mapping of critical transcription factors and their authentic binding sites within the *Hdc* promoter and enhancers should promote identification of regulatory variants that influence allergy susceptibility and severity. Today, Bill Paul’s teaching and his large body of work on IL-4 continues to inspire our fascination with type 2 immunity.

## Author Contributions

All authors contributed to the literature review and writing the paper.

## Conflict of Interest Statement

The authors declare that the research was conducted in the absence of any commercial or financial relationships that could be construed as a potential conflict of interest.

## References

[1] Zhang Y Apilado R Coleman J Ben-Sasson S Tsang S Hu-Li J Interferon gamma stabilizes the T helper cell type 1 phenotype. J Exp Med (2001) 194(2):165–72. 10.1084/jem.194.2.165 11457891 PMC2193457

[2] Zhuang Y Huang Z Nishida J Brown M Zhang L Huang H. A continuous T-bet expression is required to silence the interleukin-4-producing potential in T helper type 1 cells. Immunology (2009) 128(1):34–42. 10.1111/j.1365-2567.2009.03049.x 19689734 PMC2747137

[3] Huang H Suppressing allergic immune responses. Front Biosci (2011) 3:864–70. 10.2741/e294 21622097

[4] Qi X Hong J Chaves L Zhuang Y Chen Y Wang D Antagonistic regulation by the transcription factors C/EBPalpha and MITF specifies basophil and mast cell fates. Immunity (2013) 39(1):97–110. 10.1016/j.immuni.2013.06.012 23871207 PMC3755602

[5] Li Y Liu B Harmacek L Long Z Liang J Lukin K The transcription factors GATA2 and MITF regulate Hdc gene expression in mast cells and are required for IgE/mast cell-mediated anaphylaxis. J Allergy Clin Immunol (2017). 10.1016/j.jaci.2017.10.043 PMC6291018 29277702

[6] Simons FE. Anaphylaxis. J Allergy Clin Immunol (2010) 125(2 Suppl 2):S161–81. 10.1016/j.jaci.2009.12.981 20176258

[7] Sicherer SH Sampson HA. Food allergy. J Allergy Clin Immunol (2010) 125(2 Suppl 2):S116–25. 10.1016/j.jaci.2009.08.028 20042231

[8] Hogan SP Wang YH Strait R Finkelman FD. Food-induced anaphylaxis: mast cells as modulators of anaphylactic severity. Semin Immunopathol (2012) 34(5):643–53. 10.1007/s00281-012-0320-1 22926692 PMC3924961

[9] Vickery BP Chin S Burks AW Pathophysiology of food allergy. Pediatr Clin North Am (2011) 58(2):363–76, ix–x. 10.1016/j.pcl.2011.02.012 21453807 PMC3070117

[10] Gupta R Holdford D Bilaver L Dyer A Holl JL Meltzer D. The economic impact of childhood food allergy in the United States. JAMA Pediatr (2013) 167(11):1026–31. 10.1001/jamapediatrics.2013.2376 24042236

[11] Kemp SF Lockey RF Anaphylaxis: a review of causes and mechanisms. J Allergy Clin Immunol (2002) 110(3):341–8. 10.1067/mai.2002.126811 12209078

[12] Finkelman FD. Anaphylaxis: lessons from mouse models. J Allergy Clin Immunol (2007) 120(3):506–15; quiz 516–7. 10.1016/j.jaci.2007.07.033 17765751

[13] Strait RT Morris SC Yang M Qu XW Finkelman FD. Pathways of anaphylaxis in the mouse. J Allergy Clin Immunol (2002) 109(4):658–68. 10.1067/mai.2002.123302 11941316

[14] Reber LL Hernandez JD Galli SJ. The pathophysiology of anaphylaxis. J Allergy Clin Immunol (2017) 140(2):335–48. 10.1016/j.jaci.2017.06.003 28780941 PMC5657389

[15] Dale HH Laidlaw PP The physiological action of beta-iminazolylethylamine. J Physiol (1910) 41(5):318–44. 10.1113/jphysiol.1910.sp001406 16993030 PMC1512903

[16] Best CH Dale HH Dudley HW Thorpe WV The nature of the vaso-dilator constituents of certain tissue extracts. J Physiol (1927) 62(4):397–417. 10.1113/jphysiol.1927.sp002369 16993860 PMC1514980

[17] O’Mahony L Akdis M Akdis CA. Regulation of the immune response and inflammation by histamine and histamine receptors. J Allergy Clin Immunol (2011) 128(6):1153–62. 10.1016/j.jaci.2011.06.051 21824648

[18] Landete JM De las Rivas B Marcobal A Munoz R. Updated molecular knowledge about histamine biosynthesis by bacteria. Crit Rev Food Sci Nutr (2008) 48(8):697–714. 10.1080/10408390701639041 18756395

[19] Barcik W Pugin B Westermann P Perez NR Ferstl R Wawrzyniak M Histamine-secreting microbes are increased in the gut of adult asthma patients. J Allergy Clin Immunol (2016) 138(5):1491–4.e1497. 10.1016/j.jaci.2016.05.049 27576125

[20] Gilfillan AM Tkaczyk C. Integrated signalling pathways for mast-cell activation. Nat Rev Immunol (2006) 6(3):218–30. 10.1038/nri1782 16470226

[21] Caslin HL Kiwanuka KN Haque TT Taruselli MT MacKnight HP Paranjape A Controlling mast cell activation and homeostasis: work influenced by Bill Paul that continues today. Front Immunol (2018) 9:868. 10.3389/fimmu.2018.00868 29755466 PMC5932183

[22] Gaudenzio N Sibilano R Marichal T Starkl P Reber LL Cenac N Different activation signals induce distinct mast cell degranulation strategies. J Clin Invest (2016) 126(10):3981–98. 10.1172/JCI85538 27643442 PMC5096814

[23] Cildir G Pant H Lopez AF Tergaonkar V. The transcriptional program, functional heterogeneity, and clinical targeting of mast cells. J Exp Med (2017) 214(9):2491–506. 10.1084/jem.20170910 28811324 PMC5584128

[24] Rapanelli M Pittenger C. Histamine and histamine receptors in Tourette syndrome and other neuropsychiatric conditions. Neuropharmacology (2016) 106:85–90. 10.1016/j.neuropharm.2015.08.019 26282120

[25] Hersey SJ Sachs G Gastric acid secretion. Physiol Rev (1995) 75(1):155–89. 10.1152/physrev.1995.75.1.155 7831396

[26] McNeil BD Pundir P Meeker S Han L Undem BJ Kulka M Identification of a mast-cell-specific receptor crucial for pseudo-allergic drug reactions. Nature (2015) 519(7542):237–41. 10.1038/nature14022 25517090 PMC4359082

[27] Woolhiser MR Brockow K Metcalfe DD. Activation of human mast cells by aggregated IgG through FcgammaRI: additive effects of C3a. Clin Immunol (2004) 110(2):172–80. 10.1016/j.clim.2003.11.007 15003814

[28] Saluja R Ketelaar ME Hawro T Church MK Maurer M Nawijn MC. The role of the IL-33/IL-1RL1 axis in mast cell and basophil activation in allergic disorders. Mol Immunol (2015) 63(1):80–5. 10.1016/j.molimm.2014.06.018 25017307

[29] Yoshimoto T Tsutsui H Tominaga K Hoshino K Okamura H Akira S IL-18, although antiallergic when administered with IL-12, stimulates IL-4 and histamine release by basophils. Proc Natl Acad Sci U S A (1999) 96(24):13962–6. 10.1073/pnas.96.24.13962 10570181 PMC24173

[30] Schneider E Pollard H Lepault F Guy-Grand D Minkowski M Dy M. Histamine-producing cell-stimulating activity. Interleukin 3 and granulocyte-macrophage colony-stimulating factor induce de novo synthesis of histidine decarboxylase in hemopoietic progenitor cells. J Immunol (1987) 139(11):3710–7. 2824613

[31] Moulin D Donze O Talabot-Ayer D Mezin F Palmer G Gabay C. Interleukin (IL)-33 induces the release of pro-inflammatory mediators by mast cells. Cytokine (2007) 40(3):216–25. 10.1016/j.cyto.2007.09.013 18023358

[32] Silver MR Margulis A Wood N Goldman SJ Kasaian M Chaudhary D. IL-33 synergizes with IgE-dependent and IgE-independent agents to promote mast cell and basophil activation. Inflamm Res (2010) 59(3):207–18. 10.1007/s00011-009-0088-5 19763788

[33] Allakhverdi Z Smith DE Comeau MR Delespesse G. Cutting edge: the ST2 ligand IL-33 potently activates and drives maturation of human mast cells. J Immunol (2007) 179(4):2051–4. 10.4049/jimmunol.179.4.2051 17675461

[34] Andrade MV Iwaki S Ropert C Gazzinelli RT Cunha-Melo JR Beaven MA. Amplification of cytokine production through synergistic activation of NFAT and AP-1 following stimulation of mast cells with antigen and IL-33. Eur J Immunol (2011) 41(3):760–72. 10.1002/eji.201040718 21308681 PMC3085255

[35] Fux M Pecaric-Petkovic T Odermatt A Hausmann OV Lorentz A Bischoff SC IL-33 is a mediator rather than a trigger of the acute allergic response in humans. Allergy (2014) 69(2):216–22. 10.1111/all.12309 24205920

[36] Kubo Y Nakano K. Regulation of histamine synthesis in mouse CD4+ and CD8+ T lymphocytes. Inflamm Res (1999) 48(3):149–53. 10.1007/s000110050438 10219657

[37] Szeberenyi JB Pallinger E Zsinko M Pos Z Rothe G Orso E Inhibition of effects of endogenously synthesized histamine disturbs in vitro human dendritic cell differentiation. Immunol Lett (2001) 76(3):175–82. 10.1016/S0165-2478(01)00184-5 11306145

[38] Takamatsu S Nakashima I Nakano K Modulation of endotoxin-induced histamine synthesis by cytokines in mouse bone marrow-derived macrophages. Inflamm Res (1997) 46(Suppl 1):S91–2. 10.1007/s000110050106 9098781

[39] Takamatsu S Nakano K. Histamine synthesis by bone marrow-derived macrophages. Biosci Biotechnol Biochem (1994) 58(10):1918–9. 10.1271/bbb.58.1918 7765519

[40] Stegaev V Nies AT Porola P Mieliauskaite D Sanchez-Jimenez F Urdiales JL Histamine transport and metabolism are deranged in salivary glands in Sjogren’s syndrome. Rheumatology (Oxford) (2013) 52(9):1599–608. 10.1093/rheumatology/ket188 23709238

[41] Maslinski C Kierska D. Histamine in C3H/W mice carrying spontaneous tumors of the mammary gland. Agents Actions (1991) 33(1–2):192–4. 10.1007/BF01993164 1897438

[42] Konttinen YT Husu H Han X Passani MB Ballerini C Stegaev V Non-professional histamine producing cells, immune responses and autoimmunity. In: Stark H, editor. H4 Receptor: A Novel Drug Target in Immunoregulation and Inflammation. (2013). p. 201–58.

[43] Taguchi Y Watanabe T Kubota H Hayashi H Wada H. Purification of histidine decarboxylase from the liver of fetal rats and its immunochemical and immunohistochemical characterization. J Biol Chem (1984) 259(8):5214–21. 6425286

[44] Martin SA Bishop JO. Purification and characterization of histidine decarboxylase from mouse kidney. Biochem J (1986) 234(2):349–54. 10.1042/bj2340349 3718471 PMC1146572

[45] Ohmori E Fukui T Imanishi N Yatsunami K Ichikawa A. Purification and characterization of L-histidine decarboxylase from mouse mastocytoma P-815 cells. J Biochem (1990) 107(6):834–9. 10.1093/oxfordjournals.jbchem.a123134 2118138

[46] Joseph DR Sullivan PM Wang YM Kozak C Fenstermacher DA Behrendsen ME Characterization and expression of the complementary DNA encoding rat histidine decarboxylase. Proc Natl Acad Sci U S A (1990) 87(2):733–7. 10.1073/pnas.87.2.733 2300558 PMC53340

[47] Yamamoto J Yatsunami K Ohmori E Sugimoto Y Fukui T Katayama T cDNA-derived amino acid sequence of L-histidine decarboxylase from mouse mastocytoma P-815 cells. FEBS Lett (1990) 276(1–2):214–8. 10.1016/0014-5793(90)80545-T 2125007

[48] Furuta K Nakayama K Sugimoto Y Ichikawa A Tanaka S. Activation of histidine decarboxylase through post-translational cleavage by caspase-9 in a mouse mastocytoma P-815. J Biol Chem (2007) 282(18):13438–46. 10.1074/jbc.M609943200 17360717

[49] Komori H Nitta Y Ueno H Higuchi Y. Structural study reveals that Ser-354 determines substrate specificity on human histidine decarboxylase. J Biol Chem (2012) 287(34):29175–83. 10.1074/jbc.M112.381897 22767596 PMC3436558

[50] Ohtsu H Tanaka S Terui T Hori Y Makabe-Kobayashi Y Pejler G Mice lacking histidine decarboxylase exhibit abnormal mast cells. FEBS Lett (2001) 502(1–2):53–6. 10.1016/S0014-5793(01)02663-1 11478947

[51] Hallgren J Gurish MF. Granule maturation in mast cells: histamine in control. Eur J Immunol (2014) 44(1):33–6. 10.1002/eji.201344262 24319003

[52] Makabe-Kobayashi Y Hori Y Adachi T Ishigaki-Suzuki S Kikuchi Y Kagaya Y The control effect of histamine on body temperature and respiratory function in IgE-dependent systemic anaphylaxis. J Allergy Clin Immunol (2002) 110(2):298–303. 10.1067/mai.2002.125977 12170272

[53] Nakazawa S Sakanaka M Furuta K Natsuhara M Takano H Tsuchiya S Histamine synthesis is required for granule maturation in murine mast cells. Eur J Immunol (2014) 44(1):204–14. 10.1002/eji.201343838 24002822

[54] Kollonitsch J Perkins LM Patchett AA Doldouras GA Marburg S Duggan DE Selective inhibitors of biosynthesis of aminergic neurotransmitters. Nature (1978) 274(5674):906–8. 10.1038/274906a0 683331

[55] Kelley JL Miller CA White HL. Inhibition of histidine decarboxylase. Derivatives of histidine. J Med Chem (1977) 20(4):506–9. 10.1021/jm00214a009 850236

[56] Wu F Yu J Gehring H. Inhibitory and structural studies of novel coenzyme-substrate analogs of human histidine decarboxylase. FASEB J (2008) 22(3):890–7. 10.1096/fj.07-9566com 17965265

[57] Castellani ML Perrella A Kempuraj DJ Boucher W Tagen M Salini V Immunological activation of human umbilical cord blood mast cells induces tryptase secretion and interleukin-6, and histidine decarboxilase mRNA gene expression. Pharmacol Res (2007) 55(1):57–63. 10.1016/j.phrs.2006.10.006 17110126

[58] Chhiba KD Hsu CL Berdnikovs S Bryce PJ. Transcriptional heterogeneity of mast cells and basophils upon activation. J Immunol (2017) 198(12):4868–78. 10.4049/jimmunol.1601825 28476932 PMC5862545

[59] Nagashima Y Kako K Kim JD Fukamizu A. Enhanced histamine production through the induction of histidine decarboxylase expression by phorbol ester in Jurkat cells. Mol Med Rep (2012) 6(5):944–8. 10.3892/mmr.2012.1049 22940786 PMC3493046

[60] Galli SJ Dvorak AM Marcum JA Ishizaka T Nabel G Der Simonian H Mast cell clones: a model for the analysis of cellular maturation. J Cell Biol (1982) 95(2 Pt 1):435–44. 10.1083/jcb.95.2.435 6216259 PMC2112969

[61] Nakano T Sonoda T Hayashi C Yamatodani A Kanayama Y Yamamura T Fate of bone marrow-derived cultured mast cells after intracutaneous, intraperitoneal, and intravenous transfer into genetically mast cell-deficient W/Wv mice. Evidence that cultured mast cells can give rise to both connective tissue type and mucosal mast cells. J Exp Med (1985) 162(3):1025–43. 3897446 10.1084/jem.162.3.1025 PMC2187813

[62] Katz HR Levine JS Austen KF. Interleukin 3-dependent mouse mast cells express the cholera toxin-binding acidic glycosphingolipid, ganglioside GM1, and increase their histamine content in response to toxin. J Immunol (1987) 139(5):1640–6. 2957431

[63] Wang Z Mascarenhas N Eckmann L Miyamoto Y Sun X Kawakami T Skin microbiome promotes mast cell maturation by triggering stem cell factor production in keratinocytes. J Allergy Clin Immunol (2017) 139(4):1205–16.e1206. 10.1016/j.jaci.2016.09.019 27746235 PMC5385284

[64] Conti P Pang X Boucher W Letourneau R Reale M Barbacane RC Impact of Rantes and MCP-1 chemokines on in vivo basophilic cell recruitment in rat skin injection model and their role in modifying the protein and mRNA levels for histidine decarboxylase. Blood (1997) 89(11):4120–7. 9166854

[65] Castellani ML Ciampoli C Felaco M Tete S Conti CM Salini V Neuropeptide substance P induces mRNA expression and secretion of CXCL8 chemokine, and HDC in human umbilical cord blood mast cells. Clin Invest Med (2008) 31(6):E362–72. 10.25011/cim.v31i6.4923 19032907

[66] Tagen M Elorza A Kempuraj D Boucher W Kepley CL Shirihai OS Mitochondrial uncoupling protein 2 inhibits mast cell activation and reduces histamine content. J Immunol (2009) 183(10):6313–9. 10.4049/jimmunol.0803422 19846869 PMC2925218

[67] Kempuraj D Castellani ML Petrarca C Frydas S Conti P Theoharides TC Inhibitory effect of quercetin on tryptase and interleukin-6 release, and histidine decarboxylase mRNA transcription by human mast cell-1 cell line. Clin Exp Med (2006) 6(4):150–6. 10.1007/s10238-006-0114-7 17191106

[68] Rodriguez-Caso C Rodriguez-Agudo D Sanchez-Jimenez F Medina MA. Green tea epigallocatechin-3-gallate is an inhibitor of mammalian histidine decarboxylase. Cell Mol Life Sci (2003) 60(8):1760–3. 10.1007/s00018-003-3135-3 14521154 PMC11138632

[69] Yatsunami K Ohtsu H Tsuchikawa M Higuchi T Ishibashi K Shida A Structure of the L-histidine decarboxylase gene. J Biol Chem (1994) 269(2):1554–9. 8288622

[70] Mamune-Sato R Yamauchi K Tanno Y Ohkawara Y Ohtsu H Katayose D Functional analysis of alternatively spliced transcripts of the human histidine decarboxylase gene and its expression in human tissues and basophilic leukemia cells. Eur J Biochem (1992) 209(2):533–9. 10.1111/j.1432-1033.1992.tb17317.x 1425659

[71] Fagerberg L Hallstrom BM Oksvold P Kampf C Djureinovic D Odeberg J Analysis of the human tissue-specific expression by genome-wide integration of transcriptomics and antibody-based proteomics. Mol Cell Proteomics (2014) 13(2):397–406. 10.1074/mcp.M113.035600 24309898 PMC3916642

[72] Suzuki-Ishigaki S Numayama-Tsuruta K Kuramasu A Sakurai E Makabe Y Shimura S The mouse L-histidine decarboxylase gene: structure and transcriptional regulation by CpG methylation in the promoter region. Nucleic Acids Res (2000) 28(14):2627–33. 10.1093/nar/28.14.2627 10908316 PMC102650

[73] Yue F Cheng Y Breschi A Vierstra J Wu W Ryba T A comparative encyclopedia of DNA elements in the mouse genome. Nature (2014) 515(7527):355–64. 10.1038/nature13992 25409824 PMC4266106

[74] Hirasawa N Torigoe M Kano K Ohuchi K. Involvement of Sp1 in lipopolysaccharide-induced expression of HDC mRNA in RAW 264 cells. Biochem Biophys Res Commun (2006) 349(2):833–7. 10.1016/j.bbrc.2006.08.104 16949047

[75] Ai W Liu Y Wang TC. Yin yang 1 (YY1) represses histidine decarboxylase gene expression with SREBP-1a in part through an upstream Sp1 site. Am J Physiol Gastrointest Liver Physiol (2006) 290(6):G1096–104. 10.1152/ajpgi.00199.2005 16357063

[76] Ai W Liu Y Langlois M Wang TC. Kruppel-like factor 4 (KLF4) represses histidine decarboxylase gene expression through an upstream Sp1 site and downstream gastrin responsive elements. J Biol Chem (2004) 279(10):8684–93. 10.1074/jbc.M308278200 14670968

[77] Ling KW Ottersbach K van Hamburg JP Oziemlak A Tsai FY Orkin SH GATA-2 plays two functionally distinct roles during the ontogeny of hematopoietic stem cells. J Exp Med (2004) 200(7):871–82. 10.1084/jem.20031556 15466621 PMC2213282

[78] Lim KC Hosoya T Brandt W Ku CJ Hosoya-Ohmura S Camper SA Conditional Gata2 inactivation results in HSC loss and lymphatic mispatterning. J Clin Invest (2012) 122(10):3705–17. 10.1172/JCI61619 22996665 PMC3461906

[79] Rodrigues NP Boyd AS Fugazza C May GE Guo Y Tipping AJ GATA-2 regulates granulocyte-macrophage progenitor cell function. Blood (2008) 112(13):4862–73. 10.1182/blood-2008-01-136564 18840712

[80] Tsai FY Orkin SH. Transcription factor GATA-2 is required for proliferation/survival of early hematopoietic cells and mast cell formation, but not for erythroid and myeloid terminal differentiation. Blood (1997) 89(10):3636–43. 9160668

[81] Iwasaki H Mizuno S Arinobu Y Ozawa H Mori Y Shigematsu H The order of expression of transcription factors directs hierarchical specification of hematopoietic lineages. Genes Dev (2006) 20(21):3010–21. 10.1101/gad.1493506 17079688 PMC1620021

[82] Hayashi S McMahon AP. Efficient recombination in diverse tissues by a tamoxifen-inducible form of Cre: a tool for temporally regulated gene activation/inactivation in the mouse. Dev Biol (2002) 244(2):305–18. 10.1006/dbio.2002.0597 11944939

[83] Li Y Qi X Liu B Huang H. The STAT5-GATA2 pathway is critical in basophil and mast cell differentiation and maintenance. J Immunol (2015) 194(9):4328–38. 10.4049/jimmunol.1500018 25801432 PMC4405376

[84] Rada-Iglesias A Bajpai R Swigut T Brugmann SA Flynn RA Wysocka J. A unique chromatin signature uncovers early developmental enhancers in humans. Nature (2011) 470(7333):279–83. 10.1038/nature09692 21160473 PMC4445674

[85] Calo E Wysocka J. Modification of enhancer chromatin: what, how, and why? Mol Cell (2013) 49(5):825–37. 10.1016/j.molcel.2013.01.038 23473601 PMC3857148

[86] Heintzman ND Stuart RK Hon G Fu Y Ching CW Hawkins RD Distinct and predictive chromatin signatures of transcriptional promoters and enhancers in the human genome. Nat Genet (2007) 39(3):311–8. 10.1038/ng1966 17277777

[87] Creyghton MP Cheng AW Welstead GG Kooistra T Carey BW Steine EJ Histone H3K27ac separates active from poised enhancers and predicts developmental state. Proc Natl Acad Sci U S A (2010) 107(50):21931–6. 10.1073/pnas.1016071107 21106759 PMC3003124

[88] Spitz F Furlong EE. Transcription factors: from enhancer binding to developmental control. Nat Rev Genet (2012) 13(9):613–26. 10.1038/nrg3207 22868264

[89] Kellis M Wold B Snyder MP Bernstein BE Kundaje A Marinov GK Defining functional DNA elements in the human genome. Proc Natl Acad Sci U S A (2014) 111(17):6131–8. 10.1073/pnas.1318948111 24753594 PMC4035993

[90] Shlyueva D Stampfel G Stark A. Transcriptional enhancers: from properties to genome-wide predictions. Nat Rev Genet (2014) 15(4):272–86. 10.1038/nrg3682 24614317

[91] Koues OI Kowalewski RA Chang LW Pyfrom SC Schmidt JA Luo H Enhancer sequence variants and transcription-factor deregulation synergize to construct pathogenic regulatory circuits in B-cell lymphoma. Immunity (2015) 42(1):186–98. 10.1016/j.immuni.2014.12.021 25607463 PMC4302272

[92] May D Blow MJ Kaplan T McCulley DJ Jensen BC Akiyama JA Large-scale discovery of enhancers from human heart tissue. Nat Genet (2011) 44(1):89–93. 10.1038/ng.1006 22138689 PMC3246570

[93] Arnold CD Gerlach D Stelzer C Boryn LM Rath M Stark A. Genome-wide quantitative enhancer activity maps identified by STARR-seq. Science (2013) 339(6123):1074–7. 10.1126/science.1232542 23328393

[94] Bonn S Zinzen RP Girardot C Gustafson EH Perez-Gonzalez A Delhomme N Tissue-specific analysis of chromatin state identifies temporal signatures of enhancer activity during embryonic development. Nat Genet (2012) 44(2):148–56. 10.1038/ng.1064 22231485

[95] Krusong K Ercan-Sencicek AG Xu M Ohtsu H Anderson GM State MW High levels of histidine decarboxylase in the striatum of mice and rats. Neurosci Lett (2011) 495(2):110–4. 10.1016/j.neulet.2011.03.050 21440039 PMC3081964

[96] Rapanelli M Frick L Bito H Pittenger C. Histamine modulation of the basal ganglia circuitry in the development of pathological grooming. Proc Natl Acad Sci U S A (2017) 114(25):6599–604. 10.1073/pnas.1704547114 28584117 PMC5488951

[97] Baldan LC Williams KA Gallezot JD Pogorelov V Rapanelli M Crowley M Histidine decarboxylase deficiency causes Tourette syndrome: parallel findings in humans and mice. Neuron (2014) 81(1):77–90. 10.1016/j.neuron.2013.10.052 24411733 PMC3894588

[98] Ercan-Sencicek AG Stillman AA Ghosh AK Bilguvar K O’Roak BJ Mason CE L-histidine decarboxylase and Tourette’s syndrome. N Engl J Med (2010) 362(20):1901–8. 10.1056/NEJMoa0907006 20445167 PMC2894694

[99] Xu M Li L Ohtsu H Pittenger C. Histidine decarboxylase knockout mice, a genetic model of Tourette syndrome, show repetitive grooming after induced fear. Neurosci Lett (2015) 595:50–3. 10.1016/j.neulet.2015.03.067 25841792 PMC4424082

[100] Tanaka S Hamada K Yamada N Sugita Y Tonai S Hunyady B Gastric acid secretion in L-histidine decarboxylase-deficient mice. Gastroenterology (2002) 122(1):145–55. 10.1053/gast.2002.30312 11781289

[101] Saltzman JR Kemp JA Golner BB Pedrosa MC Dallal GE Russell RM. Effect of hypochlorhydria due to omeprazole treatment or atrophic gastritis on protein-bound vitamin B12 absorption. J Am Coll Nutr (1994) 13(6):584–91. 10.1080/07315724.1994.10718452 7706591

[102] Brogden RN Carmine AA Heel RC Speight TM Avery GS. Ranitidine: a review of its pharmacology and therapeutic use in peptic ulcer disease and other allied diseases. Drugs (1982) 24(4):267–303. 10.2165/00003495-198224040-00002 6128216

